# Anomaly Detection Technologies for Dementia Care: Monitoring Goals, Sensor Applications, and Trade-offs in Home-Based Solutions—A Narrative Review

**DOI:** 10.1177/07334648251357031

**Published:** 2025-07-08

**Authors:** Joy Lai, Bing Ye, Alex Mihailidis

**Affiliations:** 1 Faculty of Applied Science and Engineering, 7938 University of Toronto, Toronto, ON, Canada; 2 Institute of Biomedical Engineering, 7938 University of Toronto, Toronto, ON, Canada; 3 KITE, Toronto Rehabilitation Institute, Toronto, ON, Canada; 4 Department of Occupational Science and Occupational Therapy, 7938 University of Toronto, Toronto, ON, Canada

**Keywords:** dementia, caregiving, assisted living, activities of daily living (ADLs), anomaly detection, behavior monitoring, assistive technology

## Abstract

Anomaly detection technologies are increasingly used to monitor people living with dementia (PLwD) in home settings, addressing critical behaviors such as wandering, sleep disturbances, and agitation. This narrative review examines technologies used for detecting behavioral anomalies, the activities they monitor, and the trade-offs between their benefits and limitations. A systematic search across MEDLINE, IEEE Xplore, ACM Digital Library, and Web of Science identified 78 studies, categorized through thematic analysis. Three primary motivations emerged: early diagnosis, safety monitoring, and reducing caregiver stress while promoting autonomy. Technologies include GPS tracking, wearables, environmental sensors, and smart home systems, each with benefits like real-time alerts and non-intrusive monitoring but also challenges such as user compliance, false positives, and privacy concerns. While these systems enhance safety and autonomy, improving sensor accuracy, integrating AI for personalized interventions, and addressing ethical concerns are essential for long-term effectiveness and supporting the well-being of both PLwD and caregivers.

**What this paper adds**

• Broadens the Scope of Existing Research: This review offers a holistic understanding of anomaly detection technologies in dementia care, moving beyond prior studies that focus solely on ambient assisted living, algorithmic approaches, or specific behaviors like agitation.
• Comprehensive Technology Mapping: The paper systematically maps sensor technologies—such as GPS, wearables, and environmental sensors—to specific dementia-related activities. It identifies both strengths (e.g., real-time alerts from wearables) and gaps (e.g., false positives and privacy concerns) to guide future improvements.
• Real-World Relevance: By emphasizing home-based applications, it highlights the need for real-world validation often missing in prior research focused on algorithm development.

**Applications of study findings**

• Practice: Guides healthcare providers in selecting technologies suited to specific dementia-related behaviors, improving dementia care and caregiver support.
• Policy: Informs policy discussions on ethical considerations, particularly concerning privacy and data security in home-based monitoring.
• Research: Identifies gaps in long-term usability and real-world validation, encouraging future research to refine sensor technologies and develop personalized, AI-driven interventions.

## Introduction

As dementia progresses, people living with dementia (PLwD) often exhibit unusual behaviors during activities of daily living (ADLs), such as sleep disturbances, skipping meals, agitation, and wandering (Andersen et al., 2021; Fletcher-Lloyd et al., 2024; Ghaleb et al., 2022; Khan et al., 2022). These behaviors can have significant negative impacts on the well-being of both PLwD and their caregivers, underscoring the importance of detecting and monitoring them effectively. For PLwD, these behaviors may accelerate cognitive and physical decline, reduce quality of life, and increase the risk of injury or illness. For caregivers, managing these behaviors can lead to heightened stress, burnout, and emotional distress (Zwerling et al., 2016). Detecting and monitoring these dementia-related behaviors has become essential for timely intervention and improved reporting (Farsi et al., 2022; Moldovan et al., 2018). Anomaly detection technologies, such as wearable devices, environmental sensors, video surveillance, and smart home systems, have emerged as innovative methods to identify these behaviors, enabling continuous monitoring and supporting caregivers (Belmonte-Hernández et al., 2022; Khan et al., 2023; Kim et al., 2009).

Despite the advancements in this field, there remains a critical gap in comprehensively understanding the practical applications of these technologies specifically within home settings, along with their benefits and limitations. While home environments offer the comfort and familiarity that benefit PLwD, they also present unique challenges for monitoring due to their variability, unstructured settings, and the influence of caregivers or others present (Brookman et al., 2023). Previous reviews on anomaly detection technologies in home settings have largely fallen into three distinct categories: those that focus broadly on ambient assisted living technologies without centering on dementia-related behaviors (Amiribesheli et al., 2015; Cicirelli et al., 2021; Dunne et al., 2021; Mshali et al., 2018), those that emphasize algorithmic and analytical approaches to anomaly detection rather than real-world motivations (Deep et al., 2020; Dhiman & Vishwakarma, 2019; Lentzas & Vrakas, 2020; Tay et al., 2023; Wang et al., 2023), and those that are dementia-specific but primarily concerned with identifying agitation and aggression as key indicators (Khan et al., 2018). While these perspectives offer valuable insights, they do not provide a holistic understanding of the diverse motivations for dementia-specific anomaly detection—such as early diagnosis, medication adherence, and safety monitoring—or a comprehensive assessment of the technologies deployed across different ADLs in home environments.

This review aims to address the primary question: What types of technologies are used to identify dementia-related behaviors in the daily lives of PLwD who reside in home settings.

To provide a comprehensive overview, the following sub-questions guide the analysis:

(1) What motivates the use of anomaly detection, such as early diagnosis, monitoring medication adherence, and ensuring safety?
(2) What specific activities are monitored and analyzed by these technologies?
(3) What types of sensors—such as cameras, wearables, and environmental sensors—are employed for detecting anomalies in each activity?
(4) What are the benefits and limitations of these sensors for monitoring specific activities?

## Methods

### Study Rationale

This literature review provides a broad overview of anomaly detection technologies, identifying key themes and research gaps. While anomaly detection is a well-established area of research, its application to understanding the behaviors of PLwD is an ongoing area of study. Given the evolving nature of this topic, a narrative review was chosen as it best suits the research questions, allowing for the inclusion of a broad range of studies. This approach provides insights into how existing anomaly detection methodologies have been applied, their current limitations, and how they might be refined to better support dementia care. Aspects of a more structured review were incorporated, including systematic screening of the literature and thematic analysis, to enhance the rigor and reliability of the findings.

Additionally, this review contributes to an ongoing research initiative focused on advancing anomaly detection techniques for monitoring behavioral changes in PLwD. By synthesizing existing literature, it helps refine the research question and guide further investigation, ensuring that relevant advancements are considered within the study framework.

### Search Strategy

The search strategy for this narrative review was completed on August 1, 2024, with guidance from an Information Specialist at the University Health Network to ensure a thorough and systematic approach. We conducted searches in MEDLINE, IEEE Xplore, ACM Digital Library, and Web of Science using a combination of search terms related to dementia, anomaly detection, and home-based activity monitoring. To focus on relevant technological advancements, we restricted the search to English-language publications from 2000 onward.

### Screening Process

Two levels of screening were completed by a single reviewer with oversight from an experienced researcher to ensure consistency and adherence to the eligibility criteria. Covidence was used to facilitate the deduplication and screening process. The studies were assessed at two stages for eligibility: (1) title and abstract screening and (2) full-text screening.

### Eligibility Criteria

#### Inclusion Criteria

Studies must focus on adults diagnosed with any type of dementia (e.g., Alzheimer’s disease and vascular dementia) at any stage and explore anomaly detection in their daily activities (ADLs). Research involving technologies designed to support informal caregivers in monitoring anomalies is included. Participants may live at home (alone or with informal caregivers) or in community-based settings (e.g., independent senior living communities).

Eligible studies must assess anomaly detection technologies, including wearable devices (e.g., smartwatches and fitness trackers), in-home sensor systems (e.g., motion detectors and smart home systems), app-based monitoring tools, video/audio monitoring, smart appliances, or integrated multi-technology systems.

#### Exclusion Criteria

Studies were excluded if they focused primarily on professional caregivers or individuals residing in institutional or long-term care settings (e.g., nursing homes and assisted living facilities). Interventions not centered on anomaly detection were excluded, including general health monitoring devices lacking specific anomaly detection functions, non-technological approaches such as manual caregiving methods, and broad dementia management programs without a defined focus on behavioral anomaly monitoring. Additionally, studies were excluded if they lacked clear descriptions of anomaly detection methods or did not report outcomes related to anomaly detection. Outcomes not considered relevant included general health or psychological measures unrelated to technology use, purely technical performance metrics (e.g., algorithm sensitivity or specificity), and long-term health outcomes that were not explicitly linked to the effectiveness of anomaly detection technologies.

### Data Analysis

A thematic analysis was applied to the selected studies, guided by the following themes:

• Activities Monitored: Identifying daily activities detected and analyzed.
• Purpose of Detection: Determining motivations for anomaly detection (e.g., early diagnosis and safety monitoring).
• Sensors and Technologies Used: Evaluating the sensors (e.g., cameras, wearables, and environmental sensors) and technologies (e.g., smart appliances and home-based monitoring systems) employed.
• Benefits and Limitations: Analyzing qualitative advantages and constraints of the technologies and sensors.
• Impact on Quality of Life: Assessing how these technologies influence the quality of life and caregiving experience for informal caregivers.

A single reviewer conducted the qualitative analysis, manually coding the data to identify key themes and trends. Recurring patterns were consolidated, and any uncertainties in coding were resolved through iterative refinement to ensure alignment with the review’s objectives. This process was guided by an information specialist and overseen by a second reviewer to enhance rigor and consistency.

## Results

A total of 1562 references were identified from the database search. After deduplication, 631 duplicates were removed (629 automatically by Covidence and 2 manually), leaving 931 studies for title and abstract screening. Of these, 342 studies proceeded to full-text screening. Following this stage, 264 studies were excluded for the following reasons:

• A total of 206 studies involved the wrong intervention (e.g., general health monitoring devices such as pedometers or heart rate monitors without anomaly detection functionality, non-technological interventions like manual caregiving methods, or broad dementia care programs not focused on behavioral anomaly detection).
• Four studies focused on an ineligible population (e.g., professional caregivers or individuals residing in institutional settings such as nursing homes).
• Fifty-two studies lacked relevant outcomes (e.g., studies reporting psychological or social outcomes without connection to anomaly detection technologies, or evaluations of algorithms with no reference to dementia-related behaviors).
• Two studies had an inappropriate study design (e.g., protocols, commentaries, or editorial articles without original empirical data).

In total, 78 studies met the eligibility criteria and were included in this review, providing insights into the use of anomaly detection technologies for monitoring unusual dementia-related behaviors in home settings. The findings are structured according to the key research questions outlined in the introduction.

### Study Quality Considerations

Many of the included studies focused on the algorithmic development of anomaly detection rather than real-world validation. A significant portion relied on simulations or pre-existing datasets (e.g., home sensor data) instead of implementing and testing anomaly detection systems in real-life settings. This introduces potential biases, as algorithm performance in controlled or synthetic environments may not fully reflect usability, reliability, or effectiveness when deployed in home environments for PLwD.

Additionally, because the primary focus of many studies was on technological advancements, long-term usability testing and real-world trials were often not conducted. As a result, there is limited evidence on how these technologies perform over time, their acceptance by users, and their integration into daily caregiving routines.

However, the inclusion of such studies was necessary to capture the broader landscape of research in this field, as they provide valuable insights into the evolution of anomaly detection methods and their potential applications. While these studies contribute to advancing the field, future research should prioritize real-world validation to assess the practical feasibility, long-term impact, and user adoption of these technologies in dementia care. Strengthening this evidence base will be essential to ensuring that anomaly detection systems are both effective and implementable in real-world settings.

### Motivations for the Use of Anomaly Detection

This section explores the motivations behind adopting anomaly detection technologies in home settings for PLwD. It identifies common trends in their use to help others recognize research gaps and find studies aligned with their specific needs. By understanding these drivers, we aim to highlight the factors that shape the development and implementation of these systems in home care environments.

Through thematic analysis, we identified three primary motivations driving the use of anomaly detection technologies in dementia care: early diagnosis and clinical decision support, safety monitoring and intervention, and reducing caregiver stress while promoting autonomy for PLwD. While many studies address multiple motivations, only the primary focus of each study is listed in Table 1 to maintain clarity. The table offers a quick visual summary of thematic categories and corresponding studies to complement the detailed narrative analysis.

**Table 1.** Summary of the Motivations Behind Anomaly Detection.

Motivation	Studies
Early diagnosis and clinical decision support	Alam et al., 2017, 2021; AlBeiruti & Al-Begain, 2014; Ammal & Manoharan, 2023; Arifoglu et al., 2021; Arifoglu & Bouchachia, 2019; Bayahya et al., 2021; Belmonte-Fernández et al., 2020; Belmonte-Hernández et al., 2022; Bertini et al., 2021; Billis et al., 2015; Coronato & Gallo, 2012; Coronato & Paragliola, 2016; Dai et al., 2021; Fernandez-Llatas et al., 2011; Ghaleb et al., 2022; Gledson et al., 2016; Guyot et al., 2013; Husna et al., 2023; Ishii et al., 2016, 2018; Khodabandehloo & Riboni, 2021; Kim et al., 2009, 2023; Meghanani et al., 2021; Payandeh, 2016; Riboni et al., 2015; Robin et al., 2021; Robinson et al., 2021; Schinle et al., 2018; Shinkawa & Yamada, 2018a, 2018b; Solis, 2017; Sutcliffe & Sawyer, 2016; Yamsanwar et al., 2019; Zolfaghari et al., 2022, 2024
Safety monitoring and intervention	Al-Molegi & Martínez-Ballesté, 2022; Alvarez et al., 2017, 2018; Andersen et al., 2021; Avvenuti et al., 2009; Barua et al., 2020; Chen & Cheng, 2013; Enshaeifar et al., 2019; Fletcher-Lloyd et al., 2024; Fuster-Garcia et al., 2015; García-Requejo et al., 2022, 2023; Gayathri & Easwarakumar, 2016; Hao et al., 2016; Khan et al., 2022, 2023; Kim et al., 2020; Kolakowski et al., 2020; Larab et al., 2010; Lee et al., 2019; Lin et al., 2015; Parvin et al., 2018; Qiu et al., 2022; Shahid et al., 2023; Son et al., 2023; Tanaka et al., 2024; Vuong et al., 2011
Reducing caregiver stress and promoting autonomy for PLwD	Amato et al., 2018; Asghar et al., 2023; Bilodeau et al., 2014; Change et al., 2008; Chikhaoui et al., 2016, 2018; Das et al., 2016; Farsi et al., 2022; Fortin-Simard et al., 2015; Franco et al., 2010; Gao et al., 2023; Hao et al., 2016; Moldovan et al., 2018; Qadeer et al., 2022; Taleb et al., 2022

#### Early Diagnosis and Clinical Decision Support

Early detection of dementia was found to be a key goal of anomaly detection technologies, which aimed to identify cognitive decline through concerning dementia-related behaviors like memory loss, speech changes, or abnormal sleep patterns (M. A. U. Alam et al., 2021; Alam et al., 2017; AlBeiruti & Al-Begain, 2014; Ammal & Manoharan, 2023; Arifoglu et al., 2021; Arifoglu & Bouchachia, 2019a; Bayahya et al., 2021; Belmonte-Fernández et al., 2020; Belmonte-Hernández et al., 2022; Bertini et al., 2021; Billis et al., 2015; Chikhaoui et al., 2016; Coronato & Gallo, 2012; Coronato & Paragliola, 2016; Dai et al., 2021; Fernandez-Llatas et al., 2011; Ghaleb et al., 2022; Gledson et al., 2016; Guyot et al., 2013; Husna et al., 2023; Ishii et al., 2016, 2018; Khodabandehloo & Riboni, 2021; Kim et al., 2023; Kim et al., 2009; Meghanani et al., 2021; Payandeh, 2016; Riboni et al., 2015; Robin et al., 2021; Robinson et al., 2021; Schinle et al., 2018; Shinkawa & Yamada, 2018a, 2018b; Solis, 2017; Sutcliffe & Sawyer, 2016; Yamsanwar et al., 2019; Zolfaghari et al., 2022, 2024). By monitoring changes in daily behaviors and speech patterns, these tools enable timely interventions that may slow disease progression (Fernandez-Llatas et al., 2011). Anomaly detection systems also support clinical decision-making by continuously collecting data, allowing for personalized treatment plans (Gayathri & Easwarakumar, 2016). The use of artificial intelligence (AI) and machine learning further enhances precision in tracking the progression of dementia, helping caregivers identify changes in cognitive impairment (Yamsanwar et al., 2019).

#### Safety Monitoring and Intervention

Anomaly detection technologies were also found to enhance safety by detecting or preventing wandering and accidents (Al-Molegi & Martínez-Ballesté, 2022; Alvarez et al., 2017, 2018; Andersen et al., 2021; Avvenuti et al., 2009; Barua et al., 2020; Chen & Cheng, 2013; Enshaeifar et al., 2019; Fletcher-Lloyd et al., 2024; Fuster-Garcia et al., 2015; García-Requejo et al., 2022, 2023; Gayathri & Easwarakumar, 2016; Hao et al., 2016; Khan et al., 2022, 2023; Kim et al., 2020; Kolakowski et al., 2020; Larab et al., 2010; Lee et al., 2019; Lin et al., 2015; Parvin et al., 2018; Qiu et al., 2022; Shahid et al., 2023; Son et al., 2023; Tanaka et al., 2024; Vuong et al., 2011). Tools like GPS tracking, motion sensors, and smart home systems alert caregivers when PLwD stray from safe zones, enabling timely intervention (Al-Molegi & Martínez-Ballesté, 2022; Ishii et al., 2016). Fall detection and emergency response systems, using wearables and environmental sensors, provide real-time alerts, reducing injury risks and hospitalizations (Alvarez et al., 2018). By tracking nighttime hazards like restlessness and wandering, these systems can help prevent accidents and ensure greater safety during the night for PLwD (Alvarez et al., 2018). These technologies also monitor behavioral symptoms such as agitation and confusion, allowing caregivers to intervene early and prevent escalation (Chikhaoui et al., 2018).

#### Reducing Caregiver Stress and Promoting Autonomy PLwD

Another overarching motivation found was that anomaly detection technologies could reduce caregiver stress by reducing the need for constant supervision of activities, such as fall prevention, monitoring for wandering, and assistance during mealtimes (Amato et al., 2018; Asghar et al., 2023; Bilodeau et al., 2014; Change et al., 2008; Chikhaoui et al., 2016, 2018; Das et al., 2016; Farsi et al., 2022; Fortin-Simard et al., 2015; Franco et al., 2010; Gao et al., 2023; Hao et al., 2016; Moldovan et al., 2018; Qadeer et al., 2022; Taleb et al., 2022). Caregivers are alerted only when necessary, allowing them to focus on other tasks and reduce the need for constant vigilance, thereby reducing emotional and physical strain, and improving their quality of life (Das et al., 2016). Additionally, these systems promote independence for PLwD by assisting them with daily tasks, such as cooking, and medication management, by providing prompts if tasks are taking too long or steps are skipped (Taleb et al., 2022). Overall, these technologies create safe environments for PLwD while providing caregivers peace of mind and reducing burnout while improving care quality.

### Activities Monitored through Anomaly Detection

The following section delves into the specific activities and events that anomaly detection technologies aim to monitor for irregularities in PLwD. It explores how these technologies detect deviations from expected patterns in behaviors such as wandering, disruptions in daily routines, sleep disturbances, and other activities critical to the well-being and safety of individuals. By examining these targeted behaviors, this section highlights the practical applications of anomaly detection systems in identifying concerning events and ensuring timely interventions within home or community-based environments.

#### Wandering Detection

In dementia care, wandering and disorientation are common and hazardous behaviors that can greatly increase the risk of accidents or becoming lost (Andersen et al., 2021). Anomaly detection technologies that monitor mobility patterns are vital for detecting wandering in real time, enabling timely interventions to prevent harm. They enhance safety, ease caregiver anxiety, and support tracking dementia progression (Lin et al., 2015). The primary methods for detecting wandering in PLwD are GPS tracking and wearable devices (Al-Molegi & Martínez-Ballesté, 2022; Gao et al., 2023; García-Requejo et al., 2023; Lin et al., 2015; Vuong et al., 2011). Ultimately, these technologies improve the quality of life for both PLwD and caregivers by creating a safe environment and reducing the need for constant supervision. 

#### Daily Routines and Home Activities

Monitoring the completion of ADLs or daily routines is essential for understanding the cognitive and functional abilities of PLwD (Li et al., 2024). Consistently tracking ADLs can provide valuable insights into whether individuals can complete tasks independently or require prompting and support from caregivers. Environmental sensors, wearable devices, and smart home technologies were common methods used to detect anomalies in ADLs and routines (Ammal & Manoharan, 2023; Ishii et al., 2016; Shahid et al., 2023). When deviations from normal routines or difficulties in completing tasks, such as dressing, eating, or personal hygiene, are detected, it may signal the need for timely interventions and additional support (Franco et al., 2010; Ishii et al., 2016). Moreover, gradual declines in the ability to perform ADLs can be early indicators of cognitive decline, serving as a critical metric for diagnosis and ongoing monitoring of dementia progression (Ammal & Manoharan, 2023). By identifying changes in daily routines, healthcare providers and caregivers can adjust care strategies to better support the PLwD’s needs and enhance their quality of life (Parvin et al., 2018).

#### Sleeping Patterns and Resting Behavior

Sleep monitoring plays a vital role in managing and understanding dementia, as disturbances in sleep patterns are both an early indicator and a common symptom of the condition (Wong & Lovier, 2023). PLwD often suffer from insomnia, fragmented sleep, and irregular sleep-wake cycles, which can worsen cognitive decline and lead to increased agitation and behavioral issues (Rose & Lorenz, 2010). Monitoring sleep not only helps track the progression of dementia but also allows for timely interventions that improve sleep quality, enhancing the individual’s overall well-being and reducing caregiver strain (Coronato & Paragliola, 2016; Fuster-Garcia et al., 2015). Wearable devices such as actigraphy wristwatches, and environmental sensors in smart homes offer non-intrusive methods for tracking sleep-related behaviors and identifying disturbances, providing real-time data for early detection and intervention (Schinle et al., 2018). Additionally, models using synthetic data have been developed to detect sleep anomalies, further supporting caregivers by predicting issues before they escalate (Moldovan et al., 2019).

#### Instrumental Daily Activities, Social, and Cognitive Activities

Various technologies are used to detect anomalies in instrumental activities of daily living (IADLs), cognitive tasks, and social activities. These methods include speech and language processing, ambient and wearable sensors, computer-based monitoring, and virtual reality (VR) applications (Bayahya et al., 2021; Bertini et al., 2021; Yamsanwar et al., 2019). IADLs include tasks essential for independent living, such as managing finances, medication adherence, cooking, and transportation, while cognitive activities involve remembering appointments, decision-making, and problem-solving, and social activities include conversations and social engagements (Hayashi & Abe, 2019). Detecting anomalies in these areas provides valuable insights into the cognitive and functional abilities of PLwD, enabling more effective interventions by clinicians and caregivers (Meghanani et al., 2021; Riboni et al., 2015).

#### Emotional and Behavioral Deviations

Detecting behavioral anomalies such as aggression and agitation is essential for managing dementia-related symptoms (Carrarini et al., 2021). Technologies like wearables and environmental sensors track deviations in emotional responses, providing real-time insights into agitation, mood swings, and anxiety (Chikhaoui et al., 2018; Kim et al., 2023).

### Overview of Technologies and Their Benefits and Limitations

This section begins with Table 2, which outlines the technologies used in each study, organized by the specific activities they monitor as outlined in section 3.2 (e.g., wandering, daily routines, and sleep disturbances). Following the table, we discuss the key use cases for these technologies and examine their benefits and limitations, offering insights into their effectiveness and potential improvements in dementia care.

**Table 2.** Technologies Used for Monitoring Activities in Dementia Care: Sensor Types and References by Activity Category.

Sensor/Device type	References
Wandering detection	
GPS-based tracking technologies	
GPS (smartphone, mobile devices, and graph-based classification)	Al-Molegi & Martínez-Ballesté, 2022; Lin et al., 2015; Vuong et al., 2011
Wearable technologies	
Custom wearable (RAK5205: IR, GNSS, and LoRaWAN)	García-Requejo et al. (2023)
Accelerometers (tri-axial, step detection)	García-Requejo et al. (2022); Kim et al. (2009)
Triboelectric nanogenerator (BB-TENG)	Son et al. (2023)
UWB	Barua et al. (2020); Kolakowski et al. (2020)
Smart home and IoT systems	
Multimodal system (Fitbit, Intel RealSense, and Xiaomi sensors)	Belmonte-Hernández et al. (2022)
Smart home (PIR, door sensors, and RFID)	Gao et al., 2023; Ishii et al., 2018; Khodabandehloo & Riboni, 2021; Zolfaghari et al., 2022, 2024)
GNSS, infrared, and LoRaWAN for indoor/outdoor tracking	García-Requejo et al., 2023
WSNs with EEG, REM, and body movement sensors	Avvenuti et al. (2009)
Daily routines and home activities	
Environmental sensors	
PIR sensors for daily activity and sleep monitoring	Franco et al. (2010); Ishii et al. (2016)
Wearable devices	
Accelerometers and gyroscopes for ADL anomaly detection	Ammal and Manoharan (2023)
Motion and environmental sensors for ADL monitoring	Parvin et al. (2018)
Wearables for vitals and health anomaly detection	Enshaeifar et al. (2019)
Smart home and IoT systems	
Smart meters for appliance usage monitoring	Dai et al. (2021)
RFID, motion sensors, and device monitors for ADL errors	Hao et al. (2016)
Motion sensors for room stay and sleep monitoring	Shahid et al. (2023)
RFID and load sensors for kitchen activity monitoring	Fortin-Simard et al. (2015); Hao et al. (2016)
Audio and video systems	
Audio sensors and wearable cameras for ADL tracking	Guyot et al. (2013)
RGB-D cameras for object misplacement detection	Coronato and Gallo (2012)
Video sensors with PoseNet for behavior monitoring	Taleb et al. (2022)
Sleeping patterns and resting behavior	
Wearable devices	
E4 wristband for electrodermal activity, heart rate, and movement monitoring	Coronato and Paragliola (2016)
Actigraphy wristwatches for sleep-wake cycle tracking	Fuster-Garcia et al. (2015)
Accelerometers and EEG/REM sensors for sleep anomaly detection	Avvenuti et al. (2009)
Environmental sensors	
PIR sensors for sleep cycle and bedtime activity monitoring	Schinle et al. (2018)
Home-based accelerometers, gyroscopes, and infrared sensors	Belmonte-Fernández et al. (2020)
Microphones with wearable cameras for sleep preparation activities	Guyot et al. (2013)
Synthetic motion data for sleep anomaly detection with deep learning	Moldovan et al. (2019)
Instrumental daily activities, social, and cognitive activities	
Wearable and ambient sensors	
Magnetic contact, RFID, temperature, and PIR sensors for IADL monitoring	Riboni et al. (2015)
IoT-assisted system with EDA, PPG, accelerometer, and ambient sensors	M. A. U. Alam et al. (2021)
Computer and digital-based monitoring	
Speech pattern analysis for cognitive decline detection	Bertini et al. (2021); Meghanani et al. (2021); Robin et al. (2021); Shinkawa and Yamada (2018a, 2018b)
Keyboard and mouse use patterns for cognitive decline	Gledson et al. (2016); Sutcliffe and Sawyer (2016)
Email monitoring via CogLink for cognitive impairment detection	Robinson et al. (2021)
Digital tools for assessing cognitive decline in IADLs	Solis (2017)
VR for memory and visuospatial deficit assessment	Bayahya et al. (2021)
Smartphone-based cognitive tracking through app behavior	Yamsanwar et al. (2019)
Mobile apps for cognitive health monitoring with games and assessments	Husna et al. (2023)
Agitation and aggression	
Wearable sensors	
Accelerometers for detecting aggression (hitting, kicking, and pacing)	R. Alam et al. (2017); Chikhaoui et al. (2018)
Shimmer accelerometers and Kinect for aggression and wandering detection	Chikhaoui et al., 2016
Empatica E4 wristband (EDA, heart rate, accelerometer, blood volume pressure, and skin temperature) for agitation detection	Amato et al. (2018)
Lifelog sensors and smartwatches for real-time agitation/aggression monitoring	K. Kim et al. (2023)

#### GPS-Based Tracking Systems

GPS-based tracking technologies are critical for monitoring wandering behaviors in PLwD, particularly in outdoor environments. A key benefit of these technologies is their ability to provide real-time monitoring, as demonstrated by the SafeMove project (Al-Molegi & Martínez-Ballesté, 2022) and other similar systems (Lin et al., 2015; Vuong et al., 2011). These systems track the location of PLwD and send immediate alerts to caregivers when movement patterns deviate, enhancing safety through prompt intervention. However, GPS accuracy is often compromised in dense urban areas or indoors due to signal obstructions from buildings and environmental conditions (Al-Molegi & Martínez-Ballesté, 2022; Vuong et al., 2011). Additionally, these systems heavily rely on a stable internet connection; any disruption in connectivity can delay or prevent alerts from being sent, potentially putting PLwDs at risk (Al-Molegi & Martínez-Ballesté, 2022). GPS systems rely heavily on historical movement data to detect anomalies accurately, but this can be challenging when such data is unavailable or incomplete (Lin et al., 2015). These benefits and limitations highlight the importance of considering environmental and technological factors when implementing GPS-based tracking systems for monitoring wandering behaviors in PLwDs.

#### Wearable Technologies

Wearable technologies are widely used for continuous monitoring of PLwD due to their ability to track mobility, behavior, and physiological signals in real-time. Devices such as accelerometers, gyroscopes, and (Global Navigation Satellite System) GNSS sensors are instrumental in detecting wandering behaviors by generating alerts when abnormal movement patterns are identified, facilitating timely interventions (García-Requejo et al., 2023; Kim et al., 2009). These systems often leverage low-power technologies like LoRaWAN, ensuring long battery life and making wearables practical for continuous use (García-Requejo et al., 2023). Advanced devices, such as triboelectric nanogenerators (BB-TENG), further enhance monitoring capabilities by capturing specific movements related to wandering and falls, providing detailed behavioral insights (Son et al., 2023). However, wearables can face challenges with user compliance, as the discomfort of wearing multiple sensors may deter consistent usage (Kim et al., 2009; Son et al., 2023). Additionally, signal interference can reduce effectiveness, particularly when users transition between indoor and outdoor environments (García-Requejo et al., 2023).

Wearable devices play a key role in monitoring sleep patterns and resting behaviors. Wristbands and actigraphy watches provide non-intrusive tracking of sleep-wake cycles, enabling real-time care adjustments based on detected anomalies (Coronato & Paragliola, 2016; Fuster-Garcia et al., 2015). These devices support early detection of sleep disturbances while integrating seamlessly into daily routines. However, their reliance on synthetic data raises concerns about real-world applicability, requiring further testing (Moldovan et al., 2019). Additionally, wearables may struggle to distinguish between similar movements (e.g., walking vs. turning in bed) and face challenges such as data loss from device failure or non-compliance by PLwD (Coronato & Paragliola, 2016; Fuster-Garcia et al., 2015; Hao, Bouchard, et al., 2016).

Additionally, wearable technologies such as Empatica E4 wristbands and smartwatches provide valuable insights by continuously monitoring physiological and behavioral data, including heart rate and skin temperature, to detect agitation and aggression in real time (Alam et al., 2017; Amato et al., 2018; Chikhaoui et al., 2016, 2018). These real-time alerts empower caregivers to intervene promptly, reducing the risk of behavioral escalation and easing caregiver stress. However, these devices may miss subtle emotional cues and can cause discomfort with prolonged use, complicating their effectiveness (Alam et al., 2017; Amato et al., 2018; Chikhaoui et al., 2016, 2018). Wearables also face technical challenges, such as motion variability affecting data accuracy and false positives triggered by environmental factors (Avvenuti et al., 2009).

Overall, wearable technologies offer significant benefits in improving care outcomes and reducing caregiver stress through continuous monitoring and early intervention. Despite their potential, they face common challenges, including reliance on limited datasets, usability issues, and high false-positive rates in complex environments. Ongoing research is essential to refine these technologies and enhance their reliability, ensuring they remain effective tools in dementia care (Amato et al., 2018; Chikhaoui et al., 2016; Kim et al., 2020; Lee et al., 2019).

#### Environmental Sensors

Environmental sensors and smart home systems play a pivotal role in non-intrusive monitoring of PLwD, offering continuous tracking of routines and activities without requiring wearable devices. This approach makes them more comfortable for users, as sensors like PIR (Passive Infrared) sensors and Ultra-Wideband (UWB) systems track movement patterns and detect anomalies in real time (Ishii et al., 2018; Khodabandehloo & Riboni, 2021; Zolfaghari et al., 2024). These technologies provide caregivers with timely alerts; however, they can produce false positives, such as misclassifying routine activities (e.g., bathroom visits) as wandering, causing unnecessary caregiver stress (Ishii et al., 2018; Khodabandehloo & Riboni, 2021). Achieving high precision in distinguishing normal from abnormal activities is particularly challenging in complex home layouts, further impacting system accuracy.

PIR motion sensors are widely used for monitoring daily routines, enabling early detection of cognitive decline by tracking locomotion patterns, which can indicate emerging dependency or behavioral shifts (Franco et al., 2010; Zolfaghari et al., 2022). These sensors operate by detecting infrared radiation changes caused by movement, providing binary outputs that indicate the presence or absence of a person in a specific room or area (Miyazaki & Kasama, 2015). By strategically placing sensors in key locations such as doorways, hallways, and commonly used rooms, these systems can track movement frequency, transitions between spaces, and time spent in different areas (Nam Ha et al., 2006). Changes in these patterns—such as reduced movement between rooms, prolonged inactivity in one location, or increased nighttime wandering—can signal cognitive decline or disruptions in routine (Franco et al., 2010; Zolfaghari et al., 2022). These systems are also cost-effective and suitable for continuous monitoring in home settings without the need for expensive hardware (Franco et al., 2010). However, PIR sensors provide only binary outputs (i.e., presence or absence of movement), limiting their ability to capture subtle behavioral changes and increasing the risk of missing critical data or misidentifying movements of pets or visitors (Franco et al., 2010; Zolfaghari et al., 2022). Additionally, environmental sensors may struggle to capture data if the individual remains stationary for long periods, leading to inaccurate assessments of routines (Franco et al., 2010; Ishii et al., 2016).

RFID (Radio Frequency Identification) and load sensors further extend the capabilities of smart home systems by tracking interactions with appliances and household objects. These sensors monitor kitchen activities, detecting deviations from normal usage patterns and providing immediate feedback, which reduces the need for constant caregiver supervision (Fortin-Simard et al., 2015; Hao et al., 2016). For example, load sensors can detect whether a stove was left on or whether meal preparation was skipped, enhancing safety for PLwD (Hao et al., 2016). However, these technologies are limited in scope, focusing only on specific activities involving appliances, which restricts their ability to monitor broader ADLs, such as medication adherence or wandering (Fortin-Simard et al., 2015). They can also suffer from imprecision that results in false positives or missed detections, further complicating their effectiveness (Hao et al., 2016).

In addition to tracking activities, environmental sensors integrated into smart homes offer practical solutions for monitoring sleep patterns and identifying sleep-related anomalies. PIR sensors and infrared sensors, for example, can detect wake-up and bedtime routines, providing caregivers with insights into disruptions that may indicate behavioral changes or cognitive decline (Schinle et al., 2018). These systems are particularly affordable and well-suited for low-resource settings, allowing remote caregivers to monitor sleep disturbances without constant supervision (Belmonte-Fernández et al., 2020; Schinle et al., 2018). However, sensors may experience detection delays when dealing with variable datasets, impacting their ability to provide timely alerts (Belmonte-Fernández et al., 2020). Additionally, coverage limitations and the need for labeled data can hinder the accuracy of these systems in detecting subtle behavioral shifts (Belmonte-Fernández et al., 2020; Schinle et al., 2018).

Audio-based sensors, often integrated with wearable cameras, offer further support in monitoring sleep-related activities by capturing sound patterns (Guyot et al., 2013). However, these systems may struggle with overlapping sounds or brief events, leading to inaccuracies in behavior detection (Guyot et al., 2013). Advanced technologies leveraging big data and deep learning models (e.g., Random Forest or Sequential Models) show promise in improving anomaly detection, though these models often require real-world validation beyond synthetic datasets (Moldovan et al., 2019).

Overall, environmental sensors and smart home technologies provide long-term, non-disruptive monitoring, making them ideal for proactive dementia care (Avvenuti et al., 2009; Billis et al., 2015; Coronato & Paragliola, 2016; Fuster-Garcia et al., 2015; Schinle et al., 2018). However, their effectiveness depends on data quality, sensor accuracy, and real-world validation to address issues such as false positives, sensor misplacement, and privacy concerns. Ongoing efforts are needed to optimize these systems for more reliable and impactful use in dementia care (Billis et al., 2015; Coronato & Paragliola, 2016; Fuster-Garcia et al., 2015; Moldovan et al., 2019).

#### Smart Homes and IoT-Integrated Systems

Multimodal smart home and IoT systems offer a comprehensive solution by integrating wearable devices, environmental sensors, and AI algorithms to monitor both indoor and outdoor activities (Avvenuti et al., 2009; Belmonte-Hernández et al., 2022). These systems enhance safety and promote independence for PLwD by enabling continuous monitoring without constant caregiver supervision (Avvenuti et al., 2009; Belmonte-Hernández et al., 2022). However, the complexity involved in integrating multiple sensors and ensuring real-time data processing can make them challenging to install, often requiring technical support for maintenance (Alvarez et al., 2017; Belmonte-Hernández et al., 2022). Additionally, reliance on cloud platforms for data storage introduces potential issues with latency, data security, and managing large datasets, which can complicate their effectiveness (Belmonte-Hernández et al., 2022; Zolfaghari et al., 2024).

These systems are particularly effective in smart home environments, providing a holistic view of daily routines and detecting cognitive anomalies that may indicate early cognitive decline (Dai et al., 2021; Shahid et al., 2023). Through non-intrusive monitoring, such as analyzing energy consumption patterns, they help maintain autonomy while ensuring safety (Dai et al., 2021). However, their reliability depends on sensor accuracy and the quality of training data, with variability leading to high false-positive rates that may cause caregiver fatigue (Hao, Gaboury, & Bouchard, 2016; Shahid et al., 2023).

When ambient sensors are integrated with wearables in IoT systems, they provide real-time anomaly detection without disrupting daily routines (Alam et al., 2021; Riboni et al., 2015). These setups enable accurate monitoring of cognitive impairments and support early interventions, though they require ongoing calibration and maintenance, which can limit long-term practicality (Alam et al., 2021). Additionally, sensor noise in environments with multiple inhabitants may generate false alarms, reducing system reliability (Riboni et al., 2015). Technologies like RFID sensors can be useful, but their intrusive nature may disrupt routines, and continuous data collection could raise significant privacy concerns (Alam et al., 2021; Riboni et al., 2015).

#### Audio and Video Monitoring Systems

Audio and video monitoring systems offer detailed recognition of ADLs for PLwD by capturing and analyzing audio-visual data. These systems can accurately monitor specific activities, such as handwashing or cooking, which enhances the precision of anomaly detection (Guyot et al., 2013; Taleb et al., 2022). They also reduce the need for manual observation by caregivers, alleviating their physical and psychological responsibility (Guyot et al., 2013). Despite these advantages, the use of cameras and microphones in personal spaces can be intrusive, and addressing these concerns is challenging, even with anonymization techniques (Coronato & Gallo, 2012). Environmental noise, such as overlapping sounds or external disturbances, can also compromise the accuracy of these systems, leading to potential errors in detecting anomalies in daily activities (Guyot et al., 2013).

#### Computer-Based and Digital Monitoring

Computer-based monitoring provides a non-intrusive, cost-effective approach to tracking cognitive decline through digital interactions like keyboard and mouse usage. These tools offer longitudinal tracking, revealing subtle behavioral changes over time (Gledson et al., 2016; Solis, 2017; Sutcliffe & Sawyer, 2016). However, they rely heavily on consistent computer use and large datasets to detect patterns accurately, posing challenges for individuals with irregular digital habits. Additionally, filtering meaningful data from noise requires complex analysis, which can limit real-time monitoring capabilities (Gledson et al., 2016; Robinson et al., 2021; Sutcliffe & Sawyer, 2016).

Virtual reality (VR) systems and mobile cognitive apps further expand digital monitoring by creating immersive, controlled environments for cognitive assessments. VR tools support early diagnosis by reducing the need for frequent in-person evaluations, while mobile apps allow individuals to self-monitor cognitive changes through engaging activities (Bayahya et al., 2021; Husna et al., 2023; Solis, 2017). Although both technologies are accessible alternatives to traditional assessments, specialized VR equipment can hinder adoption, and PLwDs may need time to adjust to these tools, potentially delaying benefits (Bayahya et al., 2021; Husna et al., 2023; Yamsanwar et al., 2019). Moreover, mobile apps may not capture the full complexity of cognitive decline, and further testing across diverse populations is necessary to validate their effectiveness (Husna et al., 2023; Solis, 2017).

## Discussion

### Overview of Technology Use in Dementia Care

This review examined various technologies, including wearable devices, smart home sensors, GPS tracking, and audio-visual monitoring, to detect dementia-related behavioral anomalies. These systems provide real-time alerts that enhance safety, enable early interventions, and reduce caregiver stress by supporting continuous monitoring (Guyot et al., 2013; Khodabandehloo & Riboni, 2021; Son et al., 2023). The ability to track behaviors like wandering, sleep disturbances, and agitation ensures timely responses, preventing escalation, and improving care outcomes (R. Alam et al., 2017; Amato et al., 2018; Son et al., 2023).

### Key Challenges and Limitations

Despite their potential, several challenges hinder the effectiveness of these technologies.

• Sensor Accuracy and Data Reliability:
  o GPS-based tracking, while valuable outdoors, struggles with signal interference in indoor settings, leading to reduced precision (Al-Molegi & Martínez-Ballesté, 2022; Vuong et al., 2011).
  o Wearables, though effective for continuous monitoring, face issues such as discomfort, non-compliance, and signal interference from environmental factors (K.-J. Kim et al., 2009; Son et al., 2023).
• False Positives and Data Complexity:
  o Indoor positioning systems often generate high false-positive rates, leading to caregiver fatigue (Ishii et al., 2018; Khodabandehloo & Riboni, 2021).
  o Smart home sensors can misclassify routine activities, while complex data from integrated systems require significant calibration to ensure accuracy (Belmonte-Hernández et al., 2022; Zolfaghari et al., 2024).
• Privacy and Ethical Considerations:
  o Audio and video monitoring systems raise significant privacy concerns. Balancing intrusiveness with effective monitoring is critical, especially in personal spaces, where ethical issues must be carefully managed (Avvenuti et al., 2009; Guyot et al., 2013).

### Synthetic Data and Validation Challenges

The use of synthetic datasets for testing and validation presents both opportunities and challenges. While these datasets help simulate behaviors and test algorithms, they often fail to capture the complexity of real-world scenarios, limiting the generalizability of findings (Andersen et al., 2021; Arifoglu & Bouchachia, 2019a; Yahaya et al., 2019; Zolfaghari et al., 2024). This highlights the need for larger, representative datasets that reflect the diversity of behaviors in actual care environments.

### Usability and Long-Term Impact

Long-term studies assessing the impact of these technologies on both PLwD and caregivers are limited. While many systems show short-term benefits, longitudinal research is needed to better understand their role in improving care outcomes and caregiver well-being over time (Franco et al., 2010; Kim et al., 2020; Kolakowski et al., 2020; Moldovan et al., 2019). Furthermore, the usability, accessibility, and personalization of these tools will be essential for widespread adoption (García-Requejo et al., 2022; Husna et al., 2023; Khodabandehloo & Riboni, 2021).

### The Role of AI and Future Directions

Although this review did not focus on specific anomaly detection algorithms, it is important to recognize that the effectiveness of the hardware systems discussed is closely tied to the analytical methods used to interpret their data. The integration of machine learning and AI offers significant promise for personalized dementia care, allowing for the analysis of behavioral patterns and adaptive interventions over time (Arifoglu & Bouchachia, 2019a; Enshaeifar et al., 2019; Pr et al., 2023; Schinle et al., 2018; Taleb et al., 2022; Yahaya et al., 2019). Traditional rule-based systems and threshold-based methods remain widely used for simple anomaly detection, while advanced machine learning techniques are increasingly enhancing accuracy (Belmonte-Fernández et al., 2020; Janjua et al., 2016; Payandeh, 2016; Qadeer et al., 2022). For instance, Convolutional Neural Networks (CNNs) are applied in video surveillance to detect wandering or agitation, while Recurrent Neural Networks (RNNs) and Long Short-Term Memory (LSTM) networks analyze time-series data from sensors to identify deviations in movement or routines (Arifoglu & Bouchachia, 2017, 2019b; Arifoglu et al., 2020). Unsupervised learning approaches, such as clustering and autoencoders, enable anomaly detection without requiring labeled data, and hybrid models combining multiple techniques can improve reliability (Khan et al., 2023; McClean et al., 2011; Peeters et al., 2020).

Additionally, some of the limitations discussed earlier in relation to hardware—such as false positives, misclassification of behaviors, and sensitivity to environmental changes—are also influenced by the analysis methods employed. Rule-based systems may generate high false-positive rates due to rigid predefined thresholds, while deep learning models require large, high-quality datasets to improve generalizability. Addressing these challenges will require continued advancements in both sensor technology and AI-driven analytical models.

Future efforts should focus on refining sensor technology, integrating privacy-preserving features, and developing more comprehensive datasets to enhance the effectiveness of AI-based anomaly detection systems. Additionally, improving resource-efficient AI models will be critical for ensuring practical applicability in home settings, particularly in resource-limited environments (Arifoglu et al., 2020, 2021; Khodabandehloo & Riboni, 2021; Lin et al., 2015). As the field progresses, a deeper integration of AI with hardware systems—along with a focus on reducing errors and improving adaptability—will be key to advancing real-world dementia care solutions.

### Supporting Caregivers and Enhancing Autonomy

These technologies play a dual role in supporting both caregivers and PLwD by reducing the need for constant supervision and enabling earlier interventions. This helps alleviate emotional and physical stress on caregivers while promoting independence and safety for PLwD (Coronato & Gallo, 2012; Dai et al., 2021). However, seamless integration into daily routines remains critical to avoid adding unnecessary stress (Coronato & Gallo, 2012; Dai et al., 2021).

## Conclusion

Anomaly detection technologies offer significant promise for improving dementia care, particularly in home settings where early detection and intervention are essential. Wearable devices, environmental sensors, GPS systems, and audio-visual monitoring provide real-time insights that enhance safety, improve quality of life, and reduce caregiver stress.

Despite these benefits, sensor accuracy, privacy concerns, and reliance on synthetic data for validation remain challenges. Future research should focus on real-world validation, improving sensor performance, developing comprehensive datasets, and refining AI-driven solutions to deliver more personalized, reliable care. By addressing these challenges, anomaly detection systems can be optimized to enhance autonomy and well-being for PLwD, while easing the caregiver stress.
